# Cyclic di-GMP regulates *Mycobacterium tuberculosis* resistance to ethionamide

**DOI:** 10.1038/s41598-017-06289-7

**Published:** 2017-07-19

**Authors:** Hai-Nan Zhang, Zhao-Wei Xu, He-Wei Jiang, Fan-Lin Wu, Xiang He, Yin Liu, Shu-Juan Guo, Yang Li, Li-Jun Bi, Jiao-Yu Deng, Xian-En Zhang, Sheng-Ce Tao

**Affiliations:** 1 Shanghai Center for Systems Biomedicine, Key Laboratory of Systems Biomedicine (Ministry of Education), Shanghai Jiao Tong University, Shanghai, 200240 China; 2 School of Biomedical Engineering, Shanghai Jiao Tong University, Shanghai, 200240 China; 3 State Key Laboratory of Oncogenes and Related Genes, Shanghai Jiao Tong University, Shanghai, 200240 China; 4 National Key Laboratory of Biomacromolecules, Key Laboratory of Non-Coding RNA and Key Laboratory of Protein and Peptide Pharmaceuticals, Institute of Biophysics, Chinese Academy of Sciences, Beijing, 100101 China; 5 TB Healthcare Biotechnology Co., Ltd., Foshan Guangdong, 528000 China; 6 School of Stomatology and Medicine, Foshan University, Foshan 528000 Guangdong, China; 7 Guangdong Province Key Laboratory of TB Systems Biology and Translational Medicine, Foshan, 528000 China; 8 State Key Laboratory of Virology, Wuhan Institute of Virology, Chinese Academy of Sciences, Wuhan, 430071 China

## Abstract

Tuberculosis is still on the top of infectious diseases list on both mobility and mortality, especially due to drug-resistance of *Mycobacterium tuberculosis (M.tb)*. Ethionamide (ETH) is one of effective second line anti-TB drugs, a synthetic compound similar to isoniazid (INH) structurally, with existing severe problem of ETH resistance. ETH is a prodrug, which is activated by Etha inside *M.tb*, and etha is transcriptionally repressed by Ethr. We found that c-di-GMP could bind Ethr, enhanced the binding of Ethr to the promoter of etha, and then repressed the transcription of etha, thus caused resistance of *M.tb* to ETH. Through docking analysis and *in vitro* validation, we identified that c-di-GMP binds 3 amino acids of Ethr, *i.e*., Q125, R181 and E190, while the first 2 were the major binding sites. Homology analysis showed that Ethr was highly conservative among mycobacteria. Further docking analysis showed that c-di-GMP preferentially bound proteins of TetR family at the junction hole of symmetric dimer or tetramer proteins. Our results suggest a possible drug-resistance mechanism of ETH through the regulation of Ethr by c-di-GMP.

## Introduction

Tuberculosis (TB) with its causative pathogen *Mycobacterium tuberculosis (M.tb)* has afflicted humans for millennia and remains a huge threat on human life and health. TB is one of the top 10 causes of death worldwide, with 10.4 millions of new incident TB cases, an additional 1.2 million new TB/HIV cases, and 1.4 million TB deaths, respectively, in 2015^1^.

Although there are new drugs, like PBTZ169^2^ and bedaquiline^3^, the most effective drugs used in clinic are still those developed fifty years ago, including the first-line drug isoniazide (INH), rifampicin (RFA), pyrazinimide, ethambutol, streptomycin and the second-line drug ethionamide (ETH) *etc*.^4^. However, due to variety of reasons, we are now facing severe drug resistance problem of *M.tb*. In 2015, there were an estimated 480,000 new cases of multidrug-resistant TB (MDR-TB) with 9.5% extensively drug-resistant TB (XDR-TB) and another 100,000 new cases of rifampicin-resistant TB (RR-TB)^1^. In China, there were 70,000 incident cases of MDR/RR-TB^1^. Globally, there were about 250,000 deaths caused by MDR/RR-TB^1^. Recent studies showed drug resistance is usually caused by mutations, the inhA structural gene or the inha promoter region^5–7^, *i.e*., resistance to INH caused by mutation of katG gene. Other studies showed that alternate efflux pump, which pumping drug out of cell, could be another cause of drug-resistance^8, 9^. However, these mechanisms could explain only part of all the *M.tb* drug-resistance.

ETH is structurally similar to INH, which targets InhA to inhibit mycolic acid synthesis^10, 11^. ETH is activated by the monooxygenase Etha, producing the sulfoxide metabolite^12, 13^. It has been postulated that ETH and the sulfoxide metabolite are further transformed by Etha to a metabolite acts as the final toxic compound. Activated ETH acts with NAD to inhibit InhA activity^14^. As an anti-TB drug, ETH is similarly effective with INH to cure tuberculosis. Because of its adverse effects of gastrointestinal intolerance, such as nausea, vomiting or hepatotoxicity, ETH is only a second-line drug^15, 16^. For MDR-TB, ETH is still effective^17^. ETH-resistance also exists, and the known mechanism are mutations of etha, msha, ndh, or over-expression of Ethr, which repressed etha transcription^13, 18, 19^. However, these known mechanisms are not enough to explain the resistance of *M.tb* to ETH.

The bis-(3′-5′)-cyclic dimeric guanosine monophosphate (cyclic di-GMP or c-di-GMP) was one of the most common and important bacterial second messengers. C-di-GMP was first discovered by Benziman in 1987 as an allosteric activator of the *Gluconacetobacter xylinus* cellulose synthase^20^. And c-di-GMP has been shown to play a role in a wide range of cellular functions and processes over the past 29 years, including bacterial motility, biofilms formation, cell cycle progression with transition from the motile to the sessile state, differentiation and bacterial virulence^21–27^. The abundant functions that are regulated by c-di-GMP in bacteria, must be brought by many corresponding effectors. However, only a handful of c-di-GMP effectors are known, including PilZ family protein like PilZ^28^, GGDEF I site (RXXD motif)-based proteins like transcription factor FleQ^29^, riboswitch^30, 31^, and other proteins, *i.e*., STING^32^. c-di-GMP is synthesized from two molecular of GTP by diguanylate cyclases (DGCs) with GGDEF domain and is degraded into 5′-phosphoguanylyl-(3′-5′) guanosine (pGpG) by specific phosphodiesterases (PDEs) with EAL or HD-GYP domains. The role of c-di-GMP has been studied extensively in many bacteria like *E.coli, Streptomyces, Pseudomonas fluorescens* and etc., but less in mycobacteria. Recent studies have shown that both *M.tb* and *Mycobacterium smegmatis (M.sm)* contain these enzymes, *i.e*., DGC (Rv1354c) and PDE (Rv1357c) in *M.tb* and MSMEG_2196 in *M.sm*, as a bifunctional protein with GGDEF and EAL domains^33–35^. Although previous studies showed that c-di-GMP could regulate the long-term survival of mycobacteria under conditions of nutritional starvation^36^. Whereas, there is no report on whether and how DGCs or PDEs affect ETH resistance. Our previous study that using a *M.tb* proteome chip^37^, provided a list of c-di-GMP effectors of *M.tb*. Ethr is in this list. We speculated that ETH activation may associate with c-di-GMP, through binding of Ethr.

In this study, we validated the binding between c-di-GMP and Ethr, and found that c-di-GMP enhanced the binding between Ethr and the promoter of etha. We also found that the binding of c-di-GMP to Ethr down-regulates the transcription level of etha, and causes *M.sm* more resistant to ETH. To illustrate the molecular mechanism, we identified the binding sites of c-di-GMP on Ethr, which locate the junction at the hole between two monomers of the Ethr homodimer. Thus, we provided a possible mechanism of *M.tb* resistance to ETH, which is due to the binding of c-di-GMP to Ethr, but not gene mutation.

## Results

### c-di-GMP binds Ethr of *M.tb*

**Figure 1:**
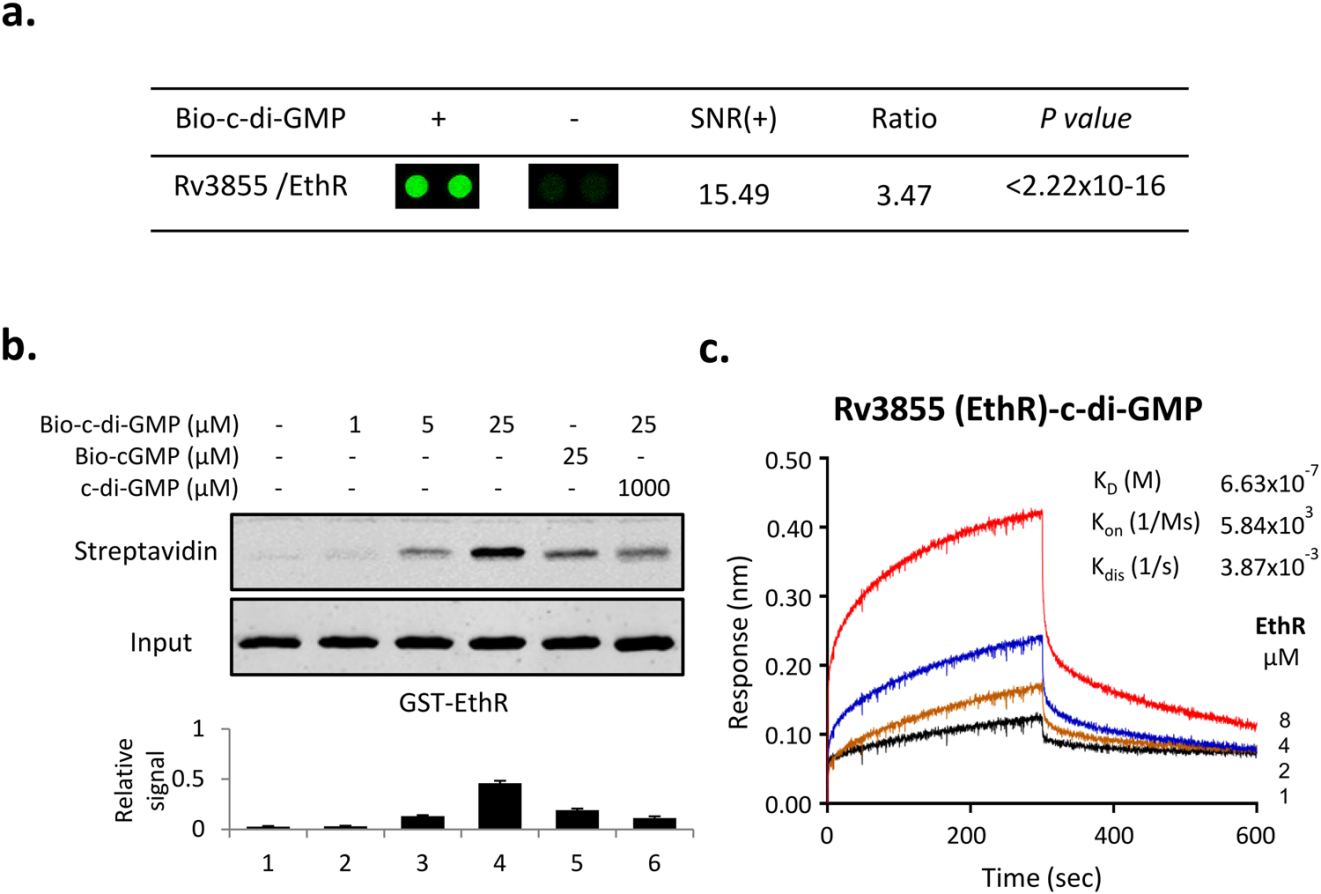
Characterization of the interaction between Ethr and c-di-GMP. (**a**). c-di-GMP binds protein Ethr on the protein microarray. Cy3 signal was measured at 532 nm by a fluorescence microarray scanner. +: in the presence of biotin-c-di-GMP; −: in the presence of biotin alone. (**b**). UV cross-linking assay for validation the interaction between Ethr and c-di-GMP. Purified GST tag Ethr was co-incubated with 1, 5, 25 μM biotinylated c-di-GMP or 25 μM biotinylated cGMP or 25 μM biotinylated c-di-GMP (Bio-c-di-GMP) with 1000 μM c-di-GMP competition. The input was visualized by a GST antibody. The relative signal to control is presented below. (**c**). Measure the binding kinetics of c-di-GMP and Ethr by Bio-Layer interference (BLI). Bio-c-di-GMP was immobilized on streptavidin coated biosenser and incubated with serially diluted Ethr. The measurement and calculation were carried out according to the manufacturer’s instruction.

By applying *M.tb* protein microarray, previously, we have identified a series of c-di-GMP binding proteins in *M.tb* ^37^. Ethr, as a transcription repressor is one of them (Fig. 1a). To further explore the possible effect of c-di-GMP to the Ethr’s function, we first set to validate and characterize the binding of c-di-GMP and Ethr. N-terminal GST-tagged Ethr was affinity purified and incubated with bio-c-di-GMP of different concentrations, *i.e*., 0, 1, 5 and 25 μM. To assure the specificity of c-di-GMP and Ethr binding, 25 μM biotinylated cGMP (bio-cGMP), and 25 μM bio-c-di-GMP with 1000 μM unlabeled c-di-GMP were also incubated with Ethr. The samples were resolved on PAGE gels and visualized by a fluorescent labeled streptavidin (Fig. 1b). The results demonstrated that the binding of c-di-GMP to Ethr is dose-dependent. Though cGMP can also bind Ethr, the binding affinity is much lower than that of c-di-GMP. Competition experiment showed that the binding of bio-c-di-GMP and Ethr could be significantly inhibited upon addition of unlabeled c-di-GMP. To rule out this possibility that the GST tag may interference with the binding, a 6× His tagged Ethr was also constructed and tested, similar results as that of GST-Ethr was obtained (Supplementary Fig. S1a). To further confirm the binding between c-di-GMP and Ethr, bio-layer interference assay (BLI)^38^ was applied to measure the binding kinetics, serially diluted Ethr were tested, consistent results were obtained among all these dilutions, and the K_D_ were determined as 0.899 μM (Fig. 1c). These results strongly indicated that c-di-GMP specifically binds Ethr with a relatively high affinity.

### c-di-GMP enhances the binding of Ethr to etha promoter

**Figure 2:**
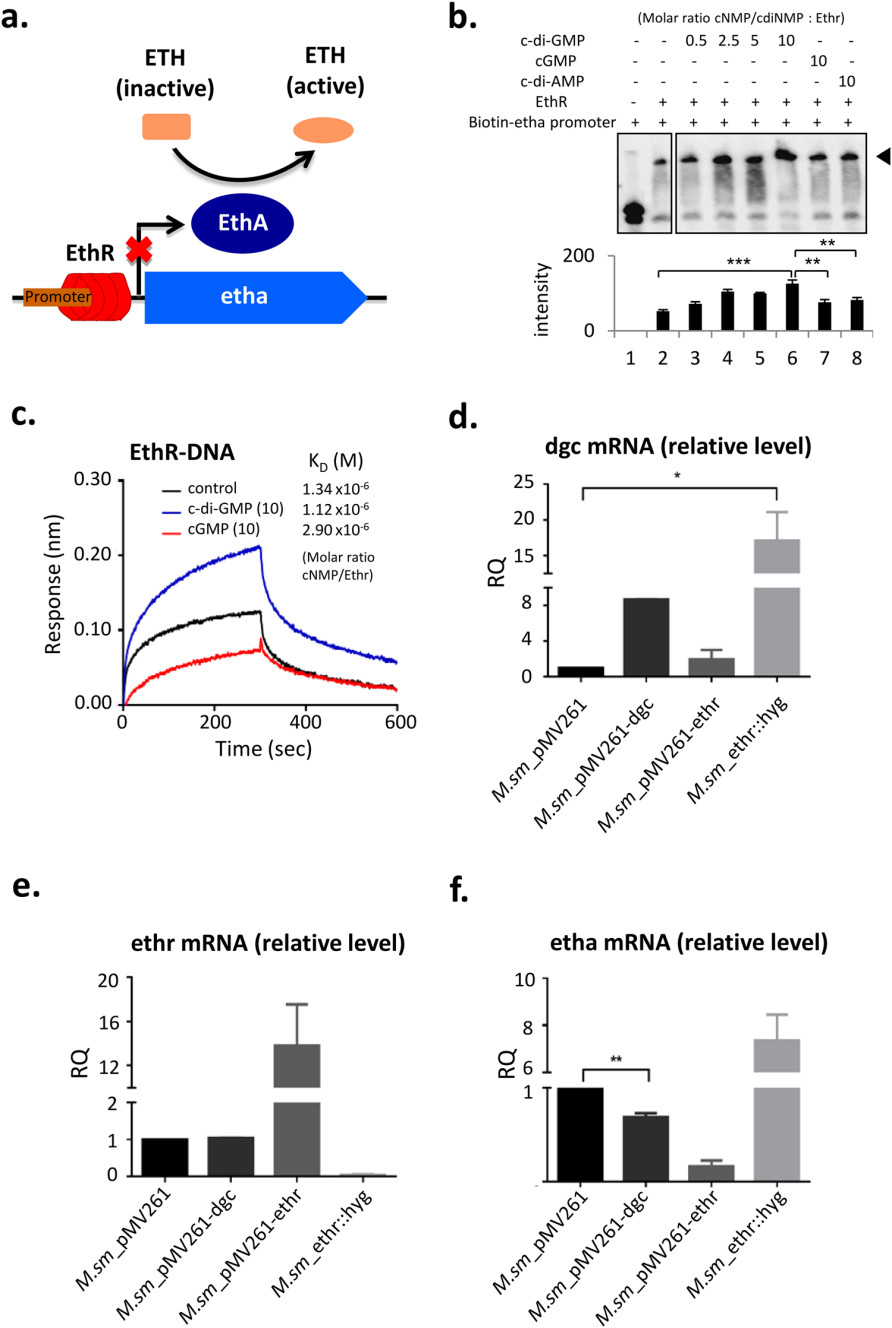
c-di-GMP enhances the binding of Ethr to the etha promoter both *in vitro* and *in vivo*. (**a**). Graphical schematic of the regulatory pathway of Ethr in mycobacteria. (**b**). EMSA assay to assess the effect of c-di-GMP to the DNA binding activity of Ethr on etha promoter. Biotinylated etha promoter was co-incubated with Ethr in presence of serially diluted c-di-GMP from 200~0 μM. High close (200 μM) of cGMP and c-di-AMP were also included as negative control. (**c**). Assess the effect of c-di-GMP to Ethr’s DNA binding activity on etha promoter by BLI assay. Biotinylated etha promoter DNA was immobilized on streptavidin coated biosensors and incubated with Ethr in the presence of 50 μM (10-fold) concentration of c-di-GMP. The cGMP was included as control. (**d**). qRT-PCR assay for determining the relative transcription level of the dgc genes. Transcription level of gene was normalized using the sigA gene as an invariant transcript. Means ± standard deviation (S.D.) represent the variant range of the data derived from three biological replicates. (**e**,**f**). qRT-PCR assay for determining the relative transcription level of the etha and ethr genes. The experiment was performed and analyzed same as that of (**d**). The *P*-values of the relative expression data were calculated by unpaired two-tailed Student’s t-test using GraphPad Prism 5. The *P*-values of the results (<0.05 or <0.01 or <0.001) are indicated by an asterisk (* or ** or ***).

It is known that Ethr, as a transcription repressor, binds to etha promoter region (DNA domain), represses the expression of etha to Etha^13, 39^ (Fig. 2a). Thus Ethr is a key regulator for the activation ETH, and may contribute to the innate resistance of mycobacteria to ETH^40^. As demonstrated by a variety of studies, inhibition of Ethr may enhance the sensitivity of mycobacteria to ETH^41–44^. Based on the validation of c-di-GMP and Ethr interaction, we speculated that c-di-GMP may interfere the DNA binding activity of Ethr. To test this speculation, Electrophoretic Mobility Shift Assay (EMSA) was carried out (Fig. 2b). Ethr was incubated with serially diluted c-di-GMPs, and cGMP or c-di-AMP were included as a negative control. The pre-incubated Ethr were then subjected for EMSA with a biotinylated double-stranded DNA derived from the etha promoter region. Compared to that of cGMP and c-di-AMP, c-di-GMP significantly enhanced the binding of Ethr to etha promoter, and this enhancement is dose-dependent (Fig. 2b). To further confirm the effect of c-di-GMP on Ethr-DNA binding, BLI assay was performed (Fig. 2c). Ethr was first incubated with c-di-GMP, and streptavidin coated sensors were prepared by adding biotinylated etha promoter DNA. Upon the addition of c-di-GMP, the K_D_ of Ethr and etha promoter DNA binding was increased from 1.34 μM to 1.12 μM. This result indicates c-di-GMP enhances Ethr and DNA binding, which is consistant with the analysis of transcription. To test the *in vivo* effect of c-di-GMP on Ethr, DGC was overexpressed in *M.sm*, named as *M.sm*-pMV261-dgc, and *M.sm* strains with ethr overexpression and knockout were included as controls, named as *M.sm*-pMV261-ethr, and *M.sm*-ethr::hyg (Table 1). qRT-PCR assay showed indeed dgc was overexpressed (Fig. 2d). Surprisingly, significant higher dgc level was also observed for *M.sm*-ethr::hyg. Furthermore, mass spectrometry analysis confirmed c-di-GMP was up-regulated in *M.sm*-pMV261-dgc (Supplementary Fig. S1b). Meanwhile, the level of ethr mRNA was not affected upon dgc overexpression (Fig. 2e). Finally, Fig. 2f showed that etha mRNA level was significantly inhibited upon the overexpression of dgc. These results indicate c-di-GMP regulates etha mRNA level through enhancing the binding of Ethr to etha promoter.

**Table 1:** Strains and plasmids used in this study.

Plasmids or Strains	Relevant genotype or features	Source
*E.coli*		
DH5a	Host for plasmid construction	TRANSGEN
BL21	Host for overexpression	TRANSGEN
*M. smegmatis mc* ^*2*^ *155*		
*M.sm*/WT	*M. smegmatis*	This study
*M.sm-*pMV261	mc^2^ 155 with pmv261	This study
*M.sm-*pMV261-ethr	mc^2^ 155 with pmv261-Ms6441	This study
*M.sm-*pMV261-dgc	mc^2^ 155 with pmv261-Ms2196	This study
*M.sm*-ethr::hyg	mc^2^ 155 Ms6441 replaced by hyg	This study
*M.sm-*ethr::hyg-pMV261	*Msm* Ms6441::hyg with pMV261	This study
*M.sm-*ethr::hyg-pMV261-ethr	*Msm* Ms6441::hyg with pMV261:: Ms6441	This study
*M.sm-*ethr::hyg-pMV261-dgc	*Msm* Ms6441::hyg with pMV261:: Ms2196	This study
pET28a(+)	Kan^r^, T7 lac promoter, N-terminal His6	This study
pET- Rv3855	Rv3855 in Nde I-Eag I sites of pET28a	This study
pMV261	Kan^r^, pAL5000 replicon	This study
pMV261- Ms6441	Ms6441 in BamHI-HindIII site of pMV261	This study
pMV261- Ms2196	Ms2196 in BamHI-HindIII site of pMV261	This study

### c-di-GMP increases *M.sm* resistance to ETH

**Figure 3:**
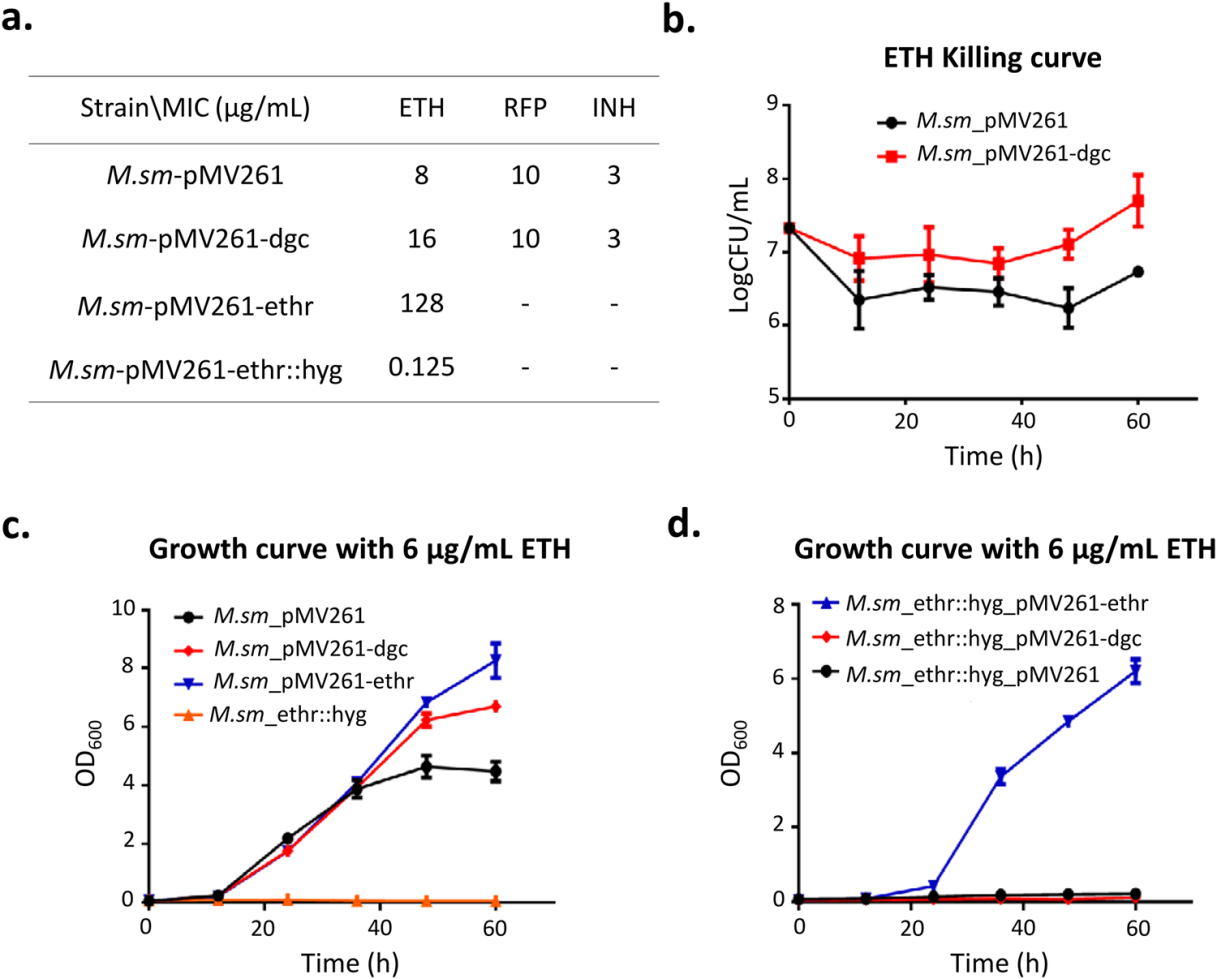
c-di-GMP increases *M.sm* resistance to ETH. (**a**). Antibioctic sensitive assay for the effects of c-di-GMP on different antibiotics. Different strains grew in serially diluted (128–0.125 μg/mL) antibiotics, *i.e*. ethionamide (ETH), rifampicin (RFP), or isoniazidea (INH). MIC represent the lowest concentration of drug under which strains can grow. (**b**). Time-Kill curve for *M.sm* during 60 h. The curve is depicted by the growth of *M.sm*-pMV261 and *M.sm*-pMV261-dgc under 120 μg/mL ETH at interval of 12 h. The concetration of ETH chosed is 15-fold MIC of *M.sm*-pMV261. (**c**,**d**). Growth curve for *M.sm* under 6 μg/mL during 60 h. The curves are depicted by the growth of *M.sm*-pMV261, *M.sm*-pMV261-dgc, *M.sm*-pMV261-ethr, and *M.sm*-ethr::hyg under 6 μg/mL ETH at interval of 12 h (**c**) and by the growth of *M.sm*-ethr::hyg-pMV261, *M.sm*-ethr::hyg-pMV261-dgc, *M.sm*-ethr::hyg-pMV261-ethr under 6 μg/mL ETH at interval of 12 h (**d**). Means ± standard deviation (S.D.) represent the variant range of the data derived from three biological replicates.

According to the results, it is highly possible that c-di-GMP affects the resistance of mycobacteria ETH. To test this, a drug sensitivity assay was performed with four strains, *i.e*., *M.sm*-pMV261, *M.sm*-pMV261-dgc, *M.sm*-pMV261-ethr, and *M.sm*-ethr::hyg (Table 1). Figure 3a showed that dgc enhanced the resistance of *M.sm* to ETH with a MIC of 16 μg/mL of *M.sm*-pMV261-dgc *vs*. 8 μg/mL that of the wild type strain *M.sm*-pMV261, while there was no drug sensitivity difference for the two strains to the two first-line antibiotics RFP and INH. Next, killing-curve assay was performed, using a ETH concentration of 15-fold MIC, *i.e*. 120 μg/mL, better growth was observed for *M.sm*-pMV261-dgc (Fig. 3b). Further, the effect of c-di-GMP was detected by determining growth curve of *M.sm* under certain amount of ETH. Firstly, in normal condition, no significant difference was observed in the growth curves among all the four strains (Supplementary Fig. S2a). With the addition of 6 μg/mL ETH, the strain *M.sm*-pMV261-dgc had an increasing tendency of growth similar to that of the positive control *M.sm*-pMV261-ethr (Fig. 3c). It is known that dgc and c-di-GMP have profound effects on the biological functions of many bacteria, and it is possible that the ETH resistance may due to factors other than Ethr. To rule out this possibility, strains with ethr knockout (*M.sm*-ethr::hyg), recovered *M.sm*-ethr::hyg-pMV261-ethr, and dgc overexpression (*M.sm*-ethr::hyg-pMV261-dgc) were constructed (Table 1). The growth of these 3 strains with 6 μg/mL ETH were monitored. Figure 3d and Fig. S2b clearly demonstrated that the hyper-sensitivity of the *M.sm*-ethr::hyg strain to ETH could only be recovered by ethr, but not dgc. These results suggest that c-di-GMP increases *M.sm* resistance to ETH depending on Ethr.

### c-di-GMP binds to the pocket between the two monomers of Ethr homodimer

**Figure 4:**
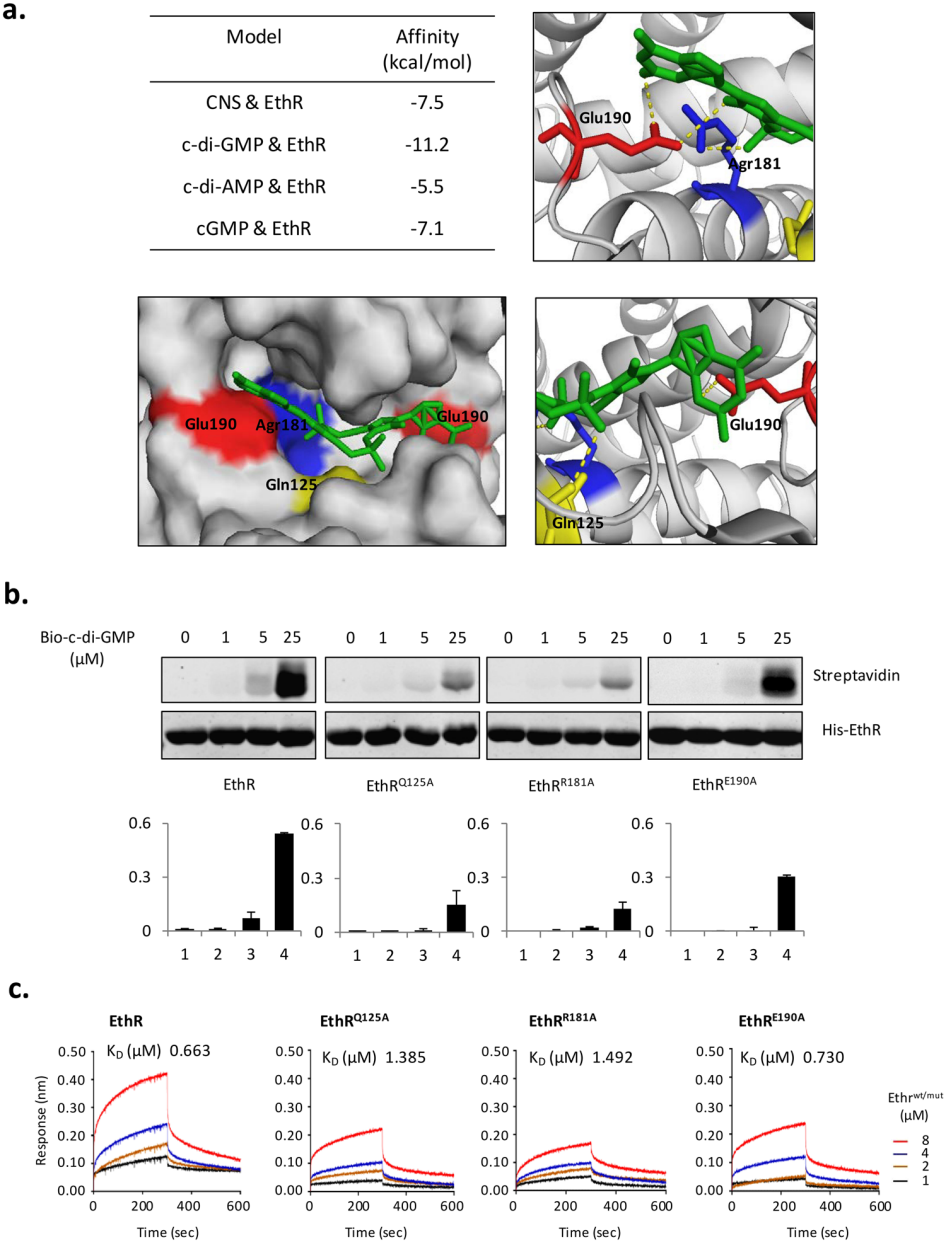
c-di-GMP binds to Ethr at Q125, R181, E190. (**a**). Docking model analysis for the binding of c-di-GMP to Ethr. Ethr was docked to c-di-GMP, c-di-AMP, cGMP, and CNS (as a positive control). The docking data is shown in the table (left). In the Ethr-c-di-GMP docking model, c-di-GMP (middle) is mediated at Watson-Crick (WC) edge of guanine bases by glutamic acid E190, and at phosphate group by arginine R181 and glutamine Q125. The PDB code of Ethr crystal structure used for the Ethr-c-di-GMP docking model is 1U9O. (**b**). UV cross-linking assay for validating the binding of Ethr mutants to c-di-GMP. Ethr and mutants of Ethr^Q125A^, Ethr^R181A^, Ethr^E190A^, were co-incubated with various concentration of biotinylation c-di-GMP. (**c**). BLI assay for validating the binding of Ethr mutants to c-di-GMP. Bio-c-di-GMP immobilized on streptavidin coated biosensor and co-incubated with various concentrations of Ethr or mutants.

To determine the binding sites of c-di-GMP in Ethr, c-di-GMP was incubated with Ethr, and subsequently was UV crosslinked. Compared with the control of Ethr alone, the binding sites were identified by mass spectrometry. The binding sites could be divided into 3 regions (Supplementary Fig. S3a and b). Docking was performed, and the highest binding affinity was obtained for region 1, *i.e*., the junction hole of the Ethr homodimer. c-di-AMP and cGMP were also docked with region 1, much lower binding affinities were obtained as compared to that of c-di-GMP (Fig. 4a). Docking analysis revealed that the possible binding sites in Ethr are Q125, R181 and E190. In the Ethr-c-di-GMP docking model, c-di-GMP is mediated at Watson-Crick (WC) edge of guanine bases by glutamic acid E190, and at the phosphate group by arginine R181 and glutamine Q125 (Fig. 4a). To confirm these three binding sites, the mutants of Q125A, R181A, E190A were constructed. Using UV cross-linking and BLI, we found that Ethr^Q125A^, Ethr^R181A^ and Ethr^E125A^ indeed had a significantly decreased binding to c-di-GMP, especially Ethr^Q125A^ and Ethr^R181A^ (Fig. 4b and c, respectively). Then we performed EMSA to detect whether c-di-GMP enhances the binding of Ethr mutants to etha promoter. Ethr and mutants were incubated with the same concentration of c-di-GMP, and then were subjected for EMSA with a biotinylated etha promoter region. Compared to Ethr, c-di-GMP barely enhanced the binding of Ethr mutants to etha promoter (Supplementary Fig. S3c). Previous study^41^ showed that inhibitors of Ethr act on helix α 4–9 of Ethr, change the distance between DNA binding domain (helix α 1–3), and affect the three-dimensional structure of Ethr, thus, inhibit its activity (Supplementary Fig. S3b). The region 1, where the three sites locate is a pocket at the interface between the two monomers of the Ethr homodimer, and is an important domain for sustaining the three-dimensional structure of Ethr (Supplementary Fig. S3a). It’s possible that the binding of c-di-GMP to the pocket could facilitate the maintenance the three-dimensional structure of Ethr homodimer to keep an appropriate distance to bind to DNA domain.

**Figure 5:**
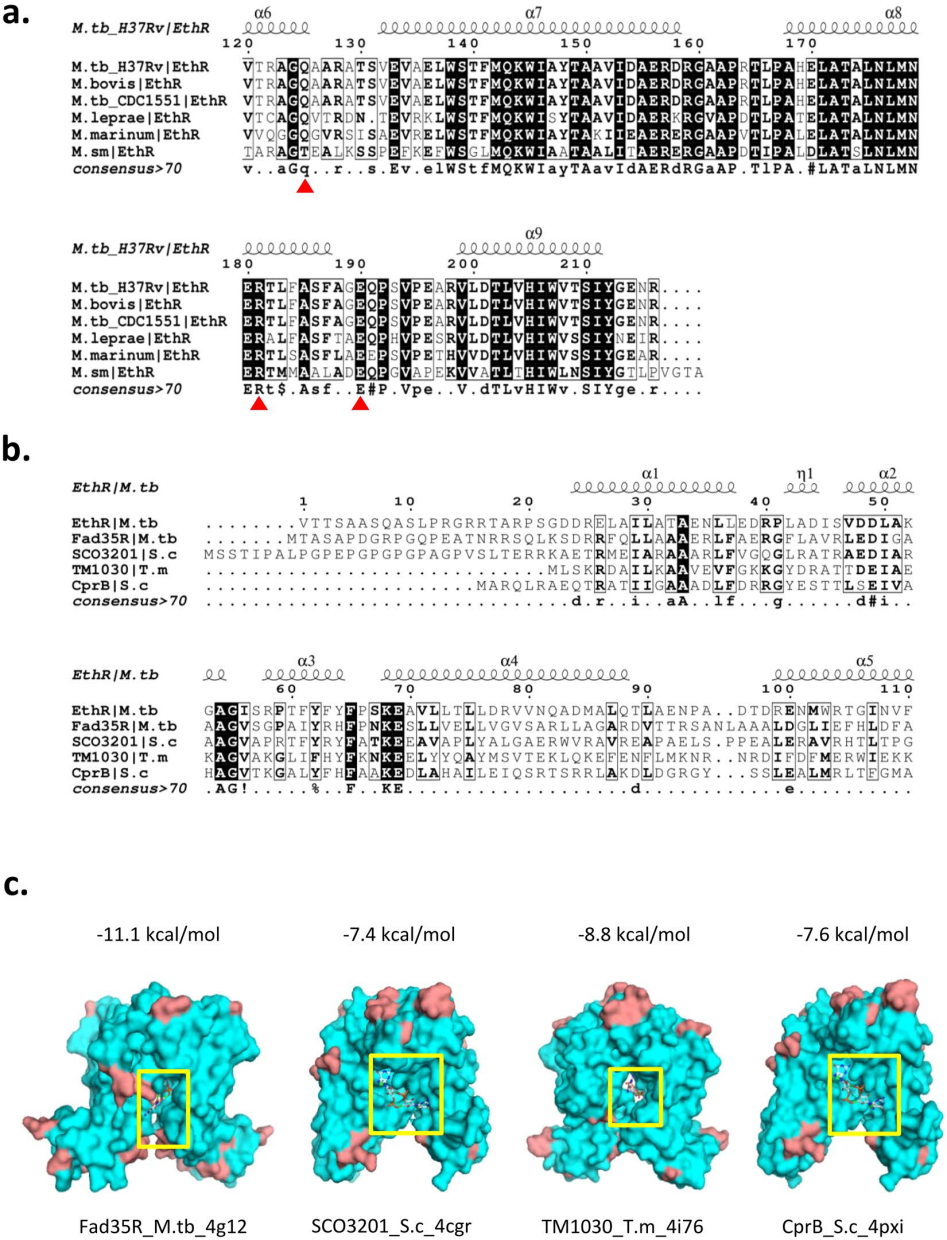
Mechanism of the binding of c-di-GMP to Ethr is general. (**a**). Sequence alignment and secondary structure element of Ethr in mycobacteria. Secondary structure element is showed above sequences. Sequence similarities are highlighted by black background (identities) and boxes (similarity). Red star represents conservative sites of Ethr with the binding of c-di-GMP at Q125, R181, E190. *M.tb*_H37Rv = *Mycobacterium tuberculosis* H37Rv, *M.bovis* = *Mycobacterium bovis*, *M.tb*_CDC1551 = *Mycobacterium tuberculosii* CDC1551, *M.leprae* = *Mycobacterium leprae*, *M.marinum* = *Mycobacterium marinum*, *M.sm* = *Mycobacterium smegmatis*. (**b**). Sequence alignment and secondary structure elements of TetR like protein in many strains. Sequence similarities are highlighted by black background (identities) and boxes (similarity). Helix α1, α2, and α3 with a higher conservation represent the DNA binding domain. *M.tb* = *Mycobacterium tuberculosis*, *S.c* = *Streptomyces coelicolor*, *T.m* = *Thermotoga maritima*. (**c**). Docking model of the four TetR like proteins with c-di-GMP. The affinity is showed above the model. Yellow boxes represent the pockets of protein that c-di-GMP binds to.

Ethr is a highly conservative protein among mycobacteria. It’s possible that c-di-GMP may also regulate other mycobacterium Ethr’s activity. We performed a sequence alignment^45^ and secondary structure element of Ethr in *Mycobacterium tuberculosis* H37Rv, *Mycobacterium bovis*, *Mycobacterium tuberculosii* CDC1551, *Mycobacterium leprae*, *Mycobacterium marinum*, and *Mycobacterium smegmatis*. The results showed that the three c-di-GMP binding sites Q125, R181, and E190 with red star mark are highly conservative among these bacteria (Fig. 5a). Ethr belongs to the TetR/CamR family, in which two paradigmatic transcriptional repressors have been characterized at the structural level: the tetracycline repressor TetR from *Escherichia coli* and the multi-drug binding protein QacR from *Staphylococcus aureus* ^18, 46^. This family had a resemble Helix-Turn-Helix domain (HTH domain), and active as a homodimer or tetramer. It’s possible that the pocket between/among the monomers of other TetR/CamR family are conservative, similar to region 1 of Ethr (Supplementary Fig. S3a), and c-di-GMP may also binds TetR/CamR family at this region. Thus, we random chose some TetR/CamR family proteins, such as *Mycobacterium tuberculosis* Fad35R, *Staphylococcus aureus* SCO3201, *Thermotoga maritima* TM1030, and *Staphylococcus aureus* CprB. We made a sequence alignment and secondary structure elements of these proteins, and docked these proteins with c-di-GMP. We found that these proteins had a similar HTH domain, consisting of α1, α2, and α3 chain (Fig. 5b), indeed could bind c-di-GMP in the pocket between homodimer, similar to that of Ethr (Fig. 5c). To further verify this observation, we randomly chose more TetR/CamR family proteins, and docked them with c-di-GMP. Those proteins are *Escherichia coli* Ycdc, *Escherichia coli* TetR, *Mycobacterium tuberculosis* KstR and *Staphylococcus aureus* QacR. And we identified that if there was a pocket no matter in the interface between homodimer or tetramer (Supplementary Fig. S4a), c-di-GMP could bind to the pocket. The affinities of these proteins docking to c-di-GMP were shown in Table 2. In addition, we incubated *Escherichia coli* Ycdc with bio-c-di-GMP, the result clearly showed that they bind each other (Supplementary Fig. S4b). These data suggests that c-di-GMP preferentially binds to the pocket region of TetR/CamR family proteins, and this is generally the case for many other bacteria.

**Table 2:** The model data list of docking of c-di-GMP to the TetR family proteins from types of microorganism.

Protein	Affinity (kcal/mol)	Pocket
TM1030_T.m	−8.8	Hole between dimer
SCO3201_S.c	−7.4	Hole between dimer
Fad35R_ M.tb	−11.1	Hole between dimer
CprB_S.c	−7.6	Hole between dimer
TetR_E.c	−7.3	Hole between dimer
QacR_S.a	−8.2	Hole between dimer
QacR_S.a	−9.5	Hole between.ramer
YcdC_E.c	−9.5	Hole between dimer
YcdC_E.c	−8.9	Hole between tetramer
KstR_M.tb	−6.7	Hole between dimer
KstR_M.tb	−9	Hole between tetramer

T.m = *Thermotoga maritime*, S.c = *Streptomyces coelicolor*, E.c = *Escherichia coli*, S.a = *Streptomyces coelicolor*, M.tb = *Mycobacterium tuberculosis*.

## Disscussion

In this study, we first confirmed the binding of c-di-GMP to Ethr. Functional analysis revealed that c-di-GMP enhances Ethr’s binding to etha promoter, and represses the transcription of etha, thus promotes the resistance of mycobacteria to ETH. Combining MS identification and docking analysis, three key c-di-GMP binding sites, *i.e*., Q125, R181, and E190 in Ethr were determined. Homolog analysis and docking model suggest that c-di-GMP may generally binds to the pocket of homodimer/tetramer of TetR/CamR family proteins.

Although several transcription factors has been known as c-di-GMP binders, such as FleQ, the first transcriptional regulator identified as c-di-GMP binder to regulate the expression of pel and other EPS genes for biofilm formation^47^, the known transcription regulators binding c-di-GMP are still limited. We identified Ethr, a TetR like transcription regulator, as a c-di-GMP binder. Our study demonstrated that c-di-GMP could directly bind to Ethr, and enhance the interaction of Ethr and etha promoter DNA to inhibit etha transcription, leading to the accumulation of inactive ETH, thus enhance the resistance of *M.sm* to ETH. ETH is only one of a thiocarbonyl-containing anti-tuberculosis medication approved for clinical use. There are two other second line anti-tuberculosis drug, such as thiocarlide, and thiacetazone, which could be similarly activated by EthA-catalyzed S-oxidation^39^. Some isolates from clinic showed a broad cross-resistance to thiocarbonyl-containing drugs including ETH, thiocarlide, and thiacetazone^39^. It’s highly possible that c-di-GMP may also enhance *M.sm* resistance to the two drugs through the Ethr-etha pathway. Although previous study showed that c-di-GMP may affect bacteria drug resistance through regulating biofilm formation^36^. However, our and others’ study demonstrate c-di-GMP doesn’t affect biofilm formation of mycobacteria (data not shown). For the first time, our results suggest a new antibiotic resistance mechanism of mycobacteria to ETH, and possibly other antibiotics.

In clinic, we already found ETH-resistance strains, with as many as 50–75% of MDR-TB isolates show ETH resistance in some population^19^. As we know, ETH resistance is associated with mutations in the etha genes, msha genes, nadh genes, inha genes, or in the Ethr overexpression strain^19, 48, 49^. Mutations in msha and ndh genes are less frequently appear, and msha encodes a glycosyltransferase involved in mycothiol biosynthesis to promote ETH activation^49^. Mutations of ndh gene result in increased intracellular NADH concentration, competitively inhibits the binding between ETH and NAD, therefore leads to resistance of ETH^49^. Because etha transcription is repressed by Ethr, thus it’s not surprising that ETH-resistance also results from overexpression of Ethr^13, 18^. Herein, we discover a new mechanism of ETH resistance, *i.e*., c-di-GMP enhances Ethr repression of etha transcription. This finding provides a possibility that we may can design small molecular to inhibit the synthesis of c-di-GMP, thus reverse the resistance of mycobacteria to ETH.

Through structure docking and biochemical experiments, we determined that Q125, R181, E190 are the binding sites of c-di-GMP to Ethr, and these sites locate at the hole between the monomers of Ethr homodimer, suggesting the hole could be the binding domain. And these three sites are highly conservative among mycobacteria, except Txxx instead of Qxxx in *M.sm*, suggesting that maybe c-di-GMP has a relatively weak binding to Ethr in *M.sm*. Although, except c-di-GMP, c-di-AMP and cGMP could also bind to Ethr with lower affinity, structurally, c-di-GMP may has the better chance to fit the “hole”.

TetR/CamR family, which had a resemble Helix-Turn-Helix domain (HTH domain), usually are active in a form of homodimer or tetramer. And most TetR/CamR family have homologous holes or pockets between/among the monomers of homodimers or tetramers. Our docking results between c-di-GMP and TetR like proteins from different bacteria show that c-di-GMP indeed could bind to these TetR like proteins, and we also identified a series of the probable binding sites (Table 2). Though we validated one candidate Ycdc indeed binds to c-di-GMP, the binding of the other proteins still need biochemical experiment to verify. These results suggest that TetR like transcription regulators could be a typical and important type of c-di-GMP receptors, through the pocket between the monomers of the dimer or tetramer. Thus, we proposed that the pocket between/among TetR like homodimer/tetramer could be a general binding site of c-di-GMP. It is known that many bacterial TetR like proteins are related to antibiotic regulation. According to the general binding of c-di-GMP to TetR like proteins, c-di-GMP may play more profound roles in bacterial drug/antibiotic resistance.

In addition, when we lift c-di-GMP level, we observed that the strain *M.sm*-pMV261-dgc had a higher survival rate in macrophage (Supplementary Fig. S5). This could be explained that except Ethr-etha pathway, c-di-GMP regulates other pathway to resist oxidation stress or nutrient deficiency in macrophage. Indeed, according to the c-di-GMP binding list^37^, there are other possible targets: wbbL2, as a rhamnosyl transferase, could regulate mycobacteria specific Rha synthetic pathway to regulate cell wall formation^50, 51^; hsaC, as a Iron-dependent extradiol dioxygenase, homologous to MSMEG_6036, regulated cholesterol metabolism to beneficial to mycobacteria persistent in macrophage^52, 53^; ephF, as a possible epoxide hydrolase, be involved in detoxification reactions following oxidative damage to lipids. We believe that c-di-GMP regulates more functions in mycobacteria. To explore the interesting functional roles of c-di-GMP, the c-di-GMP binding proteins that we identified are worth further study.

Taken together, we found that c-di-GMP binds to Ethr of 3 amino acids (Q125, R181 and E190), enhances the binding of Ethr to the promoter of etha, and causes the resistance of mycobacteria to ETH. We also proposed a general binding model of c-di-GMP to TetR like family proteins. In brief, our study not only expand the regulation roles of c-di-GMP, with the first report about regulating drug resistance, but also provide a possible mechanism of innate ETH resistance.

## Methods

### Cloning, Expression, and Purification of Ethr

The protein Ethr with GST-tag was expressed with 500 mL in SC-URA liquid medium at 30 °C, with a 2% galactose inducing when the OD_600_ reached 0.6–0.8 and then co-incubated for a further 4 h and purified as described^37^. Meanwhile, we constructed a his-tag Ethr plasmid in *E.coli* BL21. *Mycobacterium tuberculosis (M.tb) H37Rv* Ethr gene encoded by Rv3855 was amplified by PCR from a gateway entry clone carries the ethr gene using a pair of appropriate primers and cloned into the prokaryotic expression vector pET28a. E. coli BL21 was used to express the recombinant protein. Recombinant *E.coli* BL21 cells were grown in 100 mL of LB medium at 37 °C to OD_600_ = 0.6. Protein expression was induced by the addition of 1 mM isopropyl-β-d-thiogalactoside at 37 °C for 4 h. Proteins were purified on Ni-NTA affinity columns. Purified Ethr protein was stored at −80 °C.

### Validate the interaction between c-di-GMP and Ethr by western blotting

Ultraviolet (UV) cross-linking experiment with c-di-GMP were performed as described^54^. Biotin-c-di-GMP, Biotin-c-di-AMP and Biotin-c-GMP were purchased from BioLog Life Science Institute, Bremen, Germany. Ethr (1 mg/mL) was incubated with 0, 1, 5, and 25 μM Biotin-c-di-GMP in PBS buffer at RT for 1 h, followed by exposure to 254 nm UV light with 120,000 μJ from HL-2000 Hybridization Incubator (UVP, California, US) on ice for 30 mins. Meanwhile, Ethr was also co-incubated with 25 μM Biotin-c-di-AMP and 25 μM Biotin-c-GMP, reacting as described above. The proteins were electrophorized on a 10% SDS-PAGE gel, transferred to a nitrocellulose membrane. The membrane was blocked with 5% non-fat milk at RT for 1 h. After a slight washing, the membrane was probed with 1:5000 IRDye 800CW streptavidin (*LI-COR* Bioscience, Lincoln, Nebraska) at RT for 1 h, washed with TBST for 3 times. The results were recorded by a LI-COR Odyssey scanner (*LI-COR* Bioscience) and quantified by application software v3.0.21 of Odyssey infrared imaging system.

### Monitor the kinetic of c-di-GMP and Ethr interaction by BLI (Bio-Layer Interferometry)

SA tips (Pall, New York, NY) were pre-wet in 0.01 M PBS buffer (pH 7.4), which served as the background buffer for the immobilization. Octet Red 96 system from ForteBio, Pall was applied for monitoring the binding kinetics. A stable baseline (60 s) was established, and then coupled with 50 μg/ml biotin-c-di-GMP for 300 s, the non-specific binding was washed off with PBS buffer (60 s). Final immobilization levels were 0.8 ± 0.1 nm. A new baseline was established in SD buffer containing 1 × PBS, pH7.4 with 0.02% Tween-20 and 0.1% BSA. His-Ethr was prepared as a serial dilution (1, 2, 4, and 8 μM) and was allowed to bind the c-di-GMP-saturated tips for 300 s and then dissociated in SD buffer for 300 s. The results were recorded and processed by Octet Software v7.x from ForteBio system.

### Electrophoretic mobility shift assay (EMSA) for the interaction between Ethr and etha promoter

Etha promoter DNA with/without biotin-labeling for the DNA-binding activity assays were synthesized by annealing the complementary single-stranded oligo synthesized by Sangon Co., Ltd. (Sangon, Shanghai, China), and then purified by DNA purification Kit (Tiangen, Beijing, China). Ethr (0.43 μM) was incubated with 0, 2, 5, 10, 25, 50, 100, and 200 μM c-di-GMP and 200 μM c-di-AMP and 200 μM cGMP in a total volume of 13 μL PBS buffer at RT for 1 h. Then biotinylated etha promoter was added and co-incubated at RT for 20 min in a total volume of 20 μL EMSA buffer. The reaction mixtures were then subjected to 8% native PAGE, and transferred to a nylon membrane. The membrane was exposed to 254 nm UV light with 120,000 μJ from HL-2000 Hybridization Incubator (UVP, California, US) for 2 min. EMSA assay was completed using EMSA kit (Thermo) according the manufacturer’s instruction. Images were acquired using a Typhoon Scanner (GE Healthcare).

### Real-time quantitative PCR (qRT-PCR)

This experiment was performed as previously described^54^. Firstly, the wild-type (*Mycobacterium smegmatis*/*M.sm*-pMV261), dgc over-expression strain (*M.sm*-pMV261-dgc), ethr over-expression strain (*M.sm*-pMV261-ethr), and ethr-deleted mutant (*M.sm*-ethr::hyg) were constructed (Table 1). *M.sm* strains were grown and collected as described above. Total RNA was extracted according to total RNA extraction kit (Tiangen, Beijing, China). cDNA sample was obtained using a Reverse transcription kit (Promega, Madison, US). For real-time PCR analysis, each PCR (20 μL) contained 10 μL of 2× SYBR Green Master Mix Reagent (Roche, Basle, Switzerland), 1 μL of cDNA samples and 1 μL 10 μM gene-specific primers. Primers were listed in Supplementary Table S1. Expression levels of all genes were normalized to the levels of sigA gene transcripts^55^. The degrees of change in expression level were calculated using the 2^−ΔΔ^Ct method^56^.

### Antibiotics sensitive assay

*M.sm* strains were grown for 2 days in Middlebrook 7H9 media (complemented with 0.05% Tween-80 and 0.5% glycerol) containing 50 mg/ml kanamycin or 50 mg/ml hygromycin. Each culture was diluted (1:100) in 10 ml of fresh 7H9 broth for 12 h, until the OD_600_ to 0.8~1.0. Each sample was absorbed 1 μL for a dilution with 1:100, then 10 μL of the diluted sample was added on 7H10 medium plates (complemented with 0.2% glycerol) with 0, 0.0625, 0.125, 0.25, 0.5, 1, 2, 4, 8, 16, 32, 64, and 128 μg/mL antibiotics. The plates were placed in an incubator at 37 °C. After 2 days, the colony forming units were measured. In this assay, chosen three antibiotics were ethionamide, rifampicin, and isoniazid.

### Time-Kill curve assay

*M.sm* with pMV261 and pMV261-dgc was grown for 2 days in Middlebrook 7H9 media (complemented with 0.05% Tween-80 and 0.5% glycerol) containing 50 mg/ml Kan. Each culture was diluted (1:100) in 10 ml of fresh 7H9 broth to grow for 12 h, and then 120 μg/mL ethionamide (ETH) was added. Few of the cultures were absorbed and plated on 7H10 medium (complemented with 0.2% glycerol) for the determination of colony forming units as the initial point. Subsequently, the other cultures were continued to grow further at 37 °C with shaking at 200 rpm. Each 12 h until 96 h, the cultures absorbed and plated on 7H10 medium (complemented with 0.2% glycerol) for the measurement of colony forming units.

### Determination of *M.sm* growth curves and the effect of ETH

*M.sm* were grown as described above. Each culture was diluted (1:100) in 10 ml of fresh 7H9 broth with or without the 6 μg/mL ethionamide (ETH). The cultures were then allowed to grow further at 37 °C with shaking at 200 rpm. OD_600_ of every culture at each of 12 h was measured, until 96 h to stationary growth phase.

### Isolation and Quantification of c-di-GMP in *M.sm*

Isolation of c-di-GMP in *M.sm* was performed according to with modification^57^. *M.sm* strains were grown and harvested as described above. Bacterial cell pellet was obtained and are resuspended in 5 mL ddH_2_O. To extract intracellular c-di-GMP, the pellet was boiled for 10 min with buffer containing 40% methanol and 40% acetonitrile, and centrifuged at 8000 rpm for 10 min. The supernatant was transferred to a new tube. The pellets were washed one time with the same buffer. The supernatants were combined, and freeze-dried for overnight. The final pellet was resuspended in 20 μL ddH_2_O and then added 80 μL methanol. The sample was analyzed by LC-HRMS (liquid chromatography coupled high resolution mass spectrometry)^38^. LC-HRMS was performed on a Waters ACQUITY UPLC system equipped with a binary solvent delivery manager and a sample manager, coupled with a Waters Micromass Q-TOF Premier Mass Spectrometer equipped with an electrospray interface (Waters Corporation, Milford, MA). LC was performed on a BEH Amide column (50 × 2.1 mm, 1.7 µm) (Waters). The column was eluted with 5 mM Ammonium acetate (pH 9) and 95% acetonitrile in gradient mode at a flow rate of 0.30 mL/min at 30 °C.

### Structure docking model

The binding model of Ethr and c-di-GMP was analyzed using 1.0 docking software. Ethr (pdb: 1U9O) was selected for docking. The individual Ethr structure was obtained as.pdb format using the UCSF Chimera 1.10 software. The.pdb format Ethr and c-di-GMP were transformed to.pdbqt format by AutoDock Tools 1.5.6 software. Meanwhile, with this software, the docking domains were needed to set. For Ethr, the center grid box x, y, z of the three binding regions: 1, 2, 3 were set as 117.163, 17.358, 13.520; 97.136, 18.358, 0.52; 117.163, 24.358, −5.520 respectively. The size x, y, z of grid box was set as 25, 25, 25. Docking was performed with Ethr.pdbqt and c-di-GMP.pdbqt using 1.0 Docking.bat, and then the low affinity value (high affinity) was selected. Here, CNS was selected as a positive control in the docking method^42^. The 3D images of the crystal structure of Ethr were showed using Pymol^58^.

## Electronic supplementary material

Supplementary information
