# Supplementary material for: Cyclic di-GMP regulates Mycobacterium tuberculosis resistance to ethionamide
Source: Sci Rep. 2017 Jul 19;7:5860. doi: 10.1038/s41598-017-06289-7 (PMC5517500; doi:10.1038/s41598-017-06289-7)
Supplement: Supplementary file 1 — Supplementary information [file 41598_2017_6289_MOESM1_ESM.pdf]

Cyclic di-GMP regulates *Mycobacterium tuberculosis* resistance to ethionamide

Hai-Nan Zhang[1], Zhao-Wei Xu[1], He-Wei Jiang[1], Fan-lin Wu[1], Xiang He[1], Yin Liu[1], Shu-Juan Guo[1], Yang Li[1], Li-Jun Bi[4,5,6], Jiao-Yu Deng[7], Xian-En Zhang[4], and Sheng-Ce Tao[1,2,3*]

Supplementary methods

In-gel trypsin digestion. Proteins were resolved on an 10 % SDS-PAGE gel, stained with a sliver stain kit (Beyotime Institute of Biotechnology, Jiangsu, China) and excised into small gel slices. Each section was washed in water and completely destained using a mixture containing 50 % 50 mM sodium thiosulfate with 50 % 15 mM potassium ferricyanide at room temperature. Reduction was carried out with 10 mM dithiothreitol (DTT) at 60 °C for 20 min, and then with 25 mM Iodoacetimide (IAA) in the dark at room temperature for 15~20 min. Digestion was carried out using 10 μg/mL sequencing grade modified trypsin in 25 mM ammonium bicarbonate at 37 °C for 12-24 h. The supernatants were retained and the gels were sonicated twice with extraction buffer (60 % acetonitrile containing 5 % formic acid). The supernatant and the peptide extracting solution were combined, desalted and completely dried in a vacuum refrigerant centrifuge.

Nano-LC ESI-Q-TOF mass spectrometer. The peptide samples were analyzed using an ESI-Q-TOF mass spectrometer (Bruker Daltonics, Bremen, Germany) combined with an Ultimate TM 3000 nano-LC system (Dionex, Sunnyvale, CA). The dried samples were resuspended in loading buffer (2 % acetonitrile and 0.1 % formic acid), and then loaded and injected using the autosampler of the MDLC system (Michrom Bioresources Inc., Auburn, CA). Then trapped peptides were released and separated on a C18 capillary column (0.1 mm i.d.×150 mm, 3 μm, 200 Å, Michrom Bioresources Inc., Auburn, CA). The peptides were eluted with gradient 2~10 %, 10~35 %, 35~90 %, 90% B for 6 min, 34 min, 5 min, 2 min respectively with a constant flow rate of 500 nL/min. The nano-ESI-Q-TOF was set as a data dependent acquisition mode (m/z 350-1500). The Source Capillary, the flow and temperature of dry gas were set at 2000 V, 2.0 L/min and 150 °C respectively. The mass spectrometer was set as one full MS scan followed by ten MS/MS scans on the ten most intense ions from the MS spectrum. The data were analyzed using MASCOT 2.4 Peptide Mass Fingerprint (Matrix Science, Boston, MA, US) with the *M.sm* database. Search parameters were mass tolerance, 20 ppm; fragment ions, 0.05 Da. The threshold score was greater than or equal to 30, *P*-value was 0.05 and the number of peptides was more than 2 for accepting individual spectra.

THP-1 cell infection model. Human THP-1 cell line was grown in RPMI 1640 medium with 10% fetal calf serum (FBS) (Invitrogen, California, US). Cultures of the

THP-1 cells was maintained at final gross of 4 × 10^6 cells and was induced into a macrophage like state by treatment with 10 ng/mL of polymethyl acrylate (PMA) for 12 h. After removing the suspension, the adherent cells were grew for 8 h, and then infected with *M.sm* strains for 2 h at a multiplicity of infection (MOI) of 10. The contents of the culture plate wells were subsequently washed with RPMI 1640 medium and incubated in fresh complete RPMI 1640 medium with 10% FBS and 10 ng/μL gentamicin for 12, 24, 36, and 48 h. Cells were washed three times with 1× PBS and harvested for CFUs counting. Cells used for bacterial counting were lysed in 7H9 broth containing 0.05 % SDS for 10 min. Three sets of serial tenfold dilutions of the lysates from each time point were prepared in 0.05 % Tween-80, and portions were plated on 7H10 agar plates. The plates were placed in 30 °C for growth during 3-5 days.

Supplementary Figure legends

Figure 1S. Characterization of the interaction between Ethr and c-di-GMP. (a). UV cross-linking assay for validation the interaction between Ethr and c-di-GMP. Purified 6×His-Ethr was co-incubated with 1, 5, 25 μM biotinylated c-di-GMP or 25 μM biotinylated cGMP. The input was visualized by a His antibody. The relatvie signal to control is presented below. (b). UPLC-MS analysis for detecting intracellular c-di-GMP in different *M.sm* strains. Extraction and detection of mycobacterial c-di-GMP were performed as described in 'Materials and Methods' section. c-di-GMP concentration was normalized using pre-addition cGMP as an input.

Figure 2S. Growth curve for *M.sm* during 96 h. (a-b). The curves are depicted by the growth of *M.sm*-pMV261, *M.sm*-pMV261-dgc, *M.sm*-pMV261-ethr, and *M.sm*-ethr::hyg at interval of 12 h (a), and by the growth of *M.sm*-ethr::hyg-pMV261, *M.sm*-ethr::hyg-pMV261-dgc, *M.sm*-ethr::hyg-pMV261-Ethr at interval of 12 h (b). Means ± standard deviation (S.D.) represent the variant range of the data derived from three biological replicates.

Figure 3S. C-di-GMP binds to the pocket of homodimer with the highest affinity. (a). Docking model for c-di-GMP binding to three regions of Ethr dimer. Region 1 is the strongest binding domain. (b). Three dimensional structure of Ethr dimer with c-di-GMP binding in the pocket between homodimers. The exhibtion is showed from different angles. Ethr protein is composed by helix α1-α9. (c). EMSA assay to assess

the effect of c-di-GMP to the DNA binding activity of Ethr mutants on etha promoter. Biotinylated etha promoter was co-incubated with Ethr in presence of 200 μM c-di-GMP.

Figure 4S. Docking model and validation of other TetR like proteins from different bacteria with c-di-GMP. (a). Docking model of other TetR like proteins from different bacteria with c-di-GMP. Yellow boxes represent the pockets of protein that c-di-GMP binds to. E.c =*Escherichia coli,* S.a = *Streptomyces coelicolor,* M.tb = *Mycobacterium tuberculosis.* (b). UV cross-linking assay for validation the interaction between Ycdc and c-di-GMP. Purified 6×His-Ycdc was co-incubated with 5 μM Bio-c-di-GMP. Input is the same stained by ponceau.

Figure 5S. C-di-GMP enhances *M.sm* survive in macrophage of *M.sm*. Survival of strains in macrophage THP-1 cells. 4×10^6 THP-1 cells were infected in 4×10^7 *M.sm* for 12, 24, 36, 48 h. Bacteria survived in THP-1 cells were counted by CFUs. Means ± standard deviation (S.D.) of triplicate experimental samples are shown.

Supplementary Tables

Table S1 Primers used in this study

a.

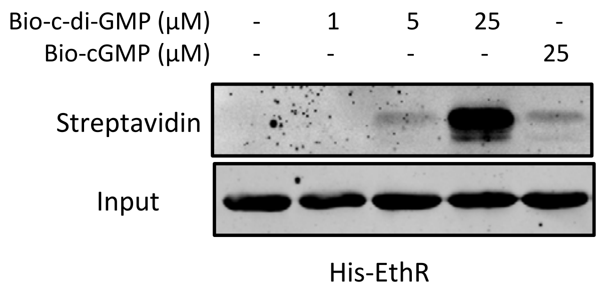

b.

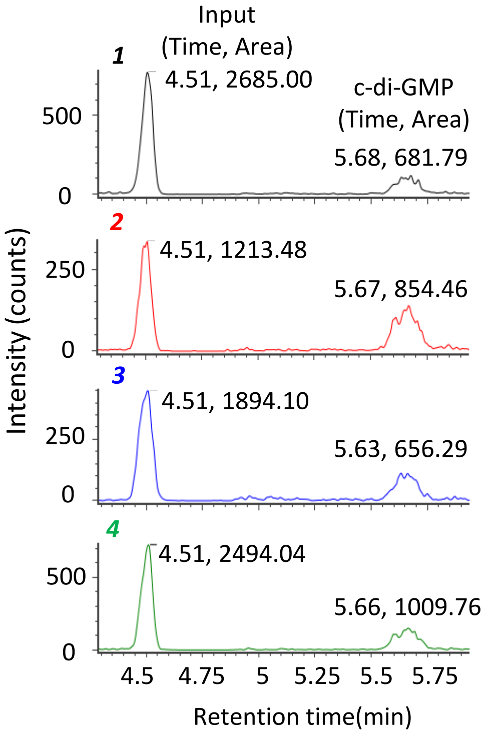

	Strains	Area Normolized (c-di-GMP/Input)
1	*M.sm*-pMV261	681.79
2	*M.sm*-pMV261- dgc	1890.62
3	*M.sm*-pMV261-ethr	930.33
4	*M.sm*-ethr::hyg	1087.07

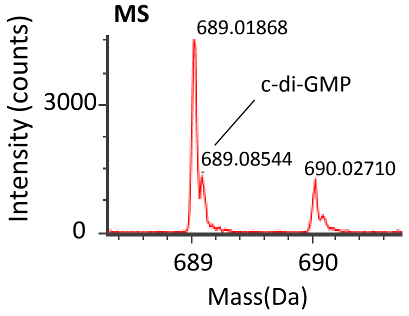

Figure S1

a.

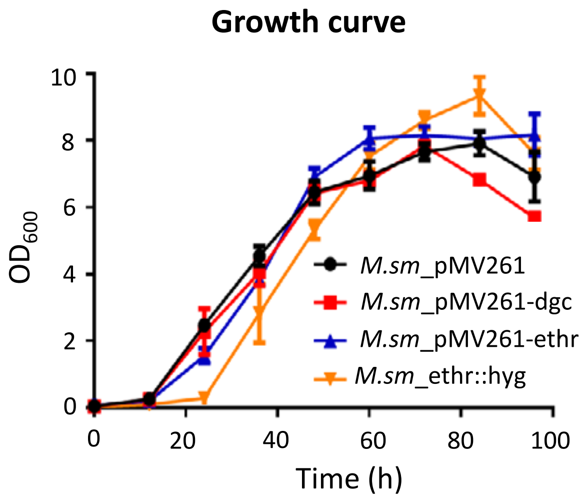

Growth curve
10
8
6
4
2
0
OD600
0
20
40
60
80
100
Time (h)
M.sm_pMV261
M.sm_pMV261-dgc
M.sm_pMV261-ethr
M.sm_ethr::hyg

b.

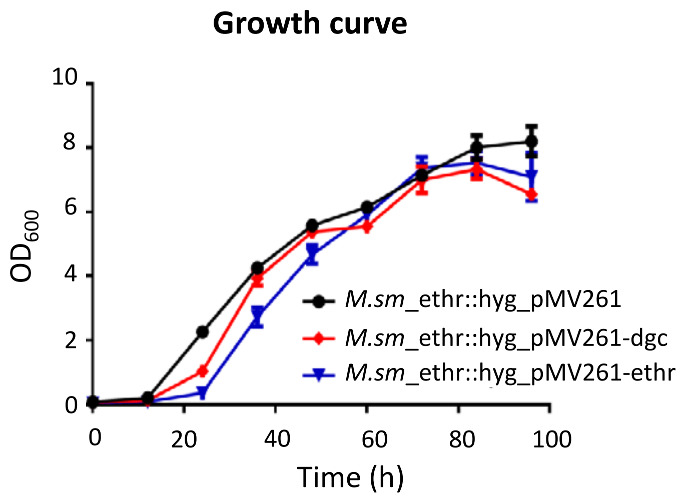

Growth curve
10
8
6
4
2
0
OD600
0
20
40
60
80
100
Time (h)
M.sm_ethr::hyg_pMV261
M.sm_ethr::hyg_pMV261-dgc
M.sm_ethr::hyg_pMV261-ethr

Figure S2

a.

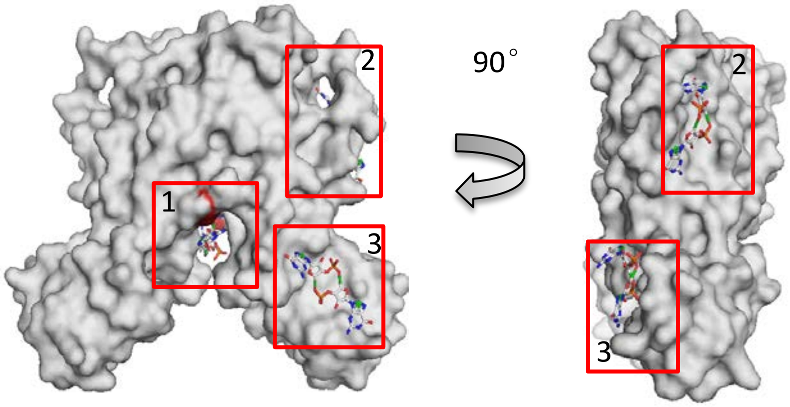

Model (c-di-GMP & EthR)	Affinity (kcal/mol)
1	-11.2
2	-8.3
3	-7.9

b.

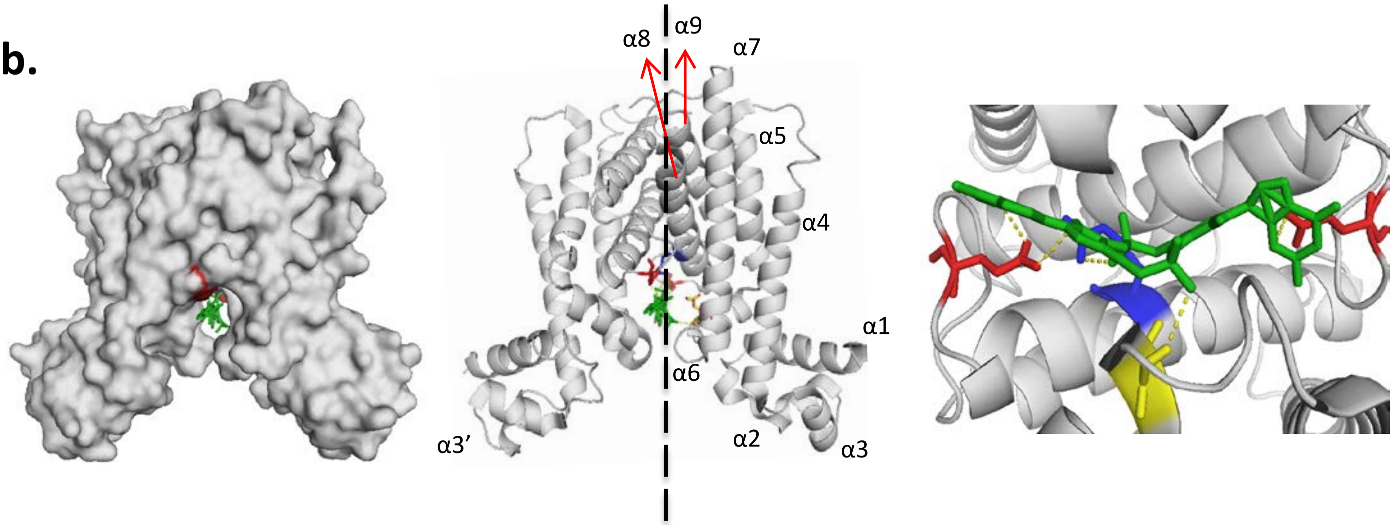

c.

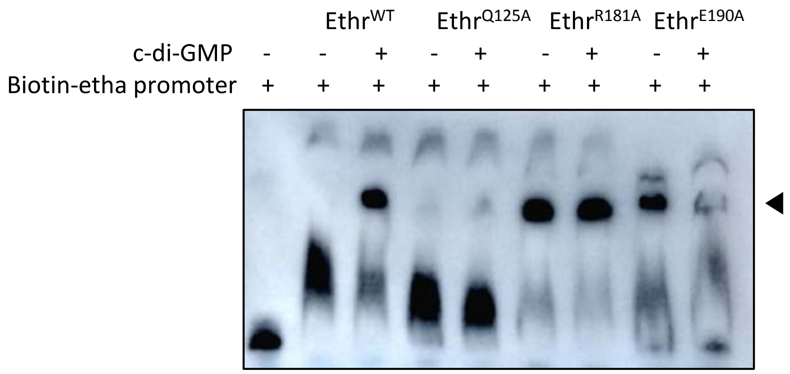

Figure S3

a.

b.

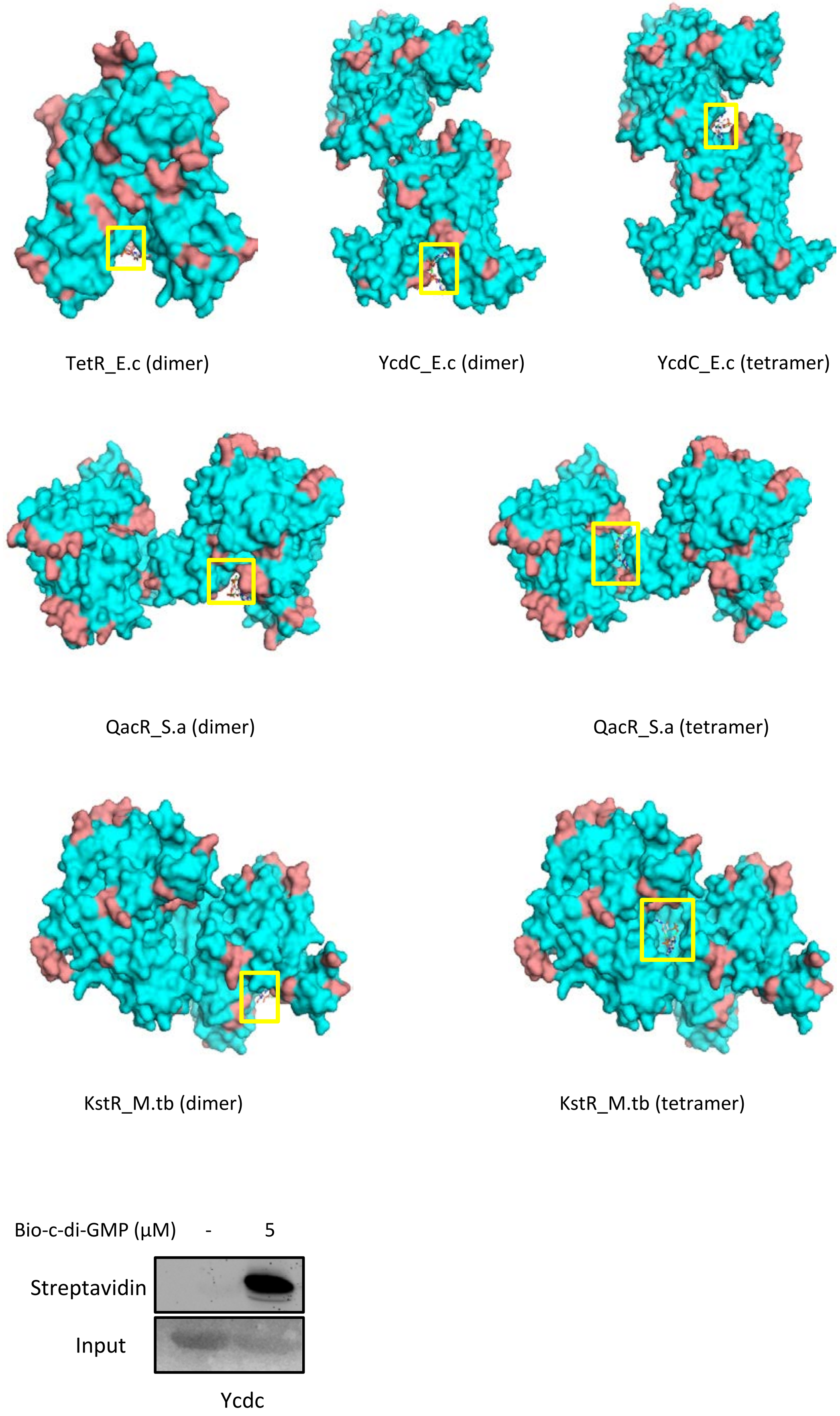

TetR_E.c (dimer)
YcdC_E.c (dimer)
YcdC_E.c (tetramer)
QacR_S.a (dimer)
QacR_S.a (tetramer)
KstR_M.tb (dimer)
KstR_M.tb (tetramer)
Bio-c-di-GMP (μM)
-
5
Streptavidin
Input
Ycdc

Figure S4

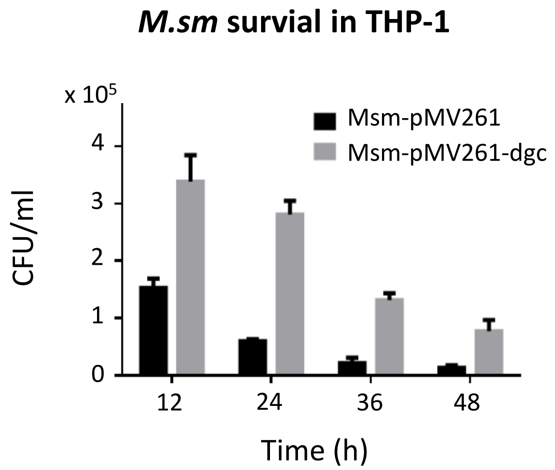
M.sm survial in THP-1
x 10^5
CFU/ml
4
3
2
1
0
Msm-pMV261
Msm-pMV261-dgc
12
24
36
48
Time (h)

Figure S5

Table S1 Primers used in this study

Name	Sequence 5'—3'	Usage
Rv3855(ethr)f	GGAATTCCATATGGTGACCACCTCCGCGGCCAGTCAGG	Clone and expression
Rv3855(ethr)r	TACCGGCCGTTAGCGGTTCTCGCCGTAAATGCTGGTC	Clone and expression
Ms6441(ethr)f	CGCGGATCCGTGACCACCGCCAGTCAGA	Clone and expression
Ms6441(ethr)r	CCCAAGCTTCTACGCCGTCCCGACGGGCAA	Clone and expression
Ms2196(dgc)f	CGCGGATCCGTGTCCGAGAGCCTGA	Clone and expression
Ms2196(dgc)r	CCCAAGCTTTCACTTGTCATCGTCGTCCTTGTAGTCAACCAGCATG CGAGGCGCA	Clone and expression
Ms6440pf	CGGTCATGGATCCACGCTATCAACGTAATGTCGAGGCCGTCAACG AGATGTCGACACTATCGA	EMSA
Ms6440pr	CTGACTGGCCGCGGAGGTGGTCACCCTGGCAGCTTACTACGTGTC GATAGATGTCGACATCTCGTTGACGG	EMSA
Ms6440pr	CTGACTGGCCGCGGAGGTGGTCACCCTGGCAGCTTACTACGTGTC GATAGATGTCGACATCTCGTTGACGG (labeled with biotin)	EMSA
sigAf	AAAAACCATCTGCTGGAGGC	Reverse transcription -PCR
sigAr	TAGTCGAACTTCTCGACCGC	Reverse transcription -PCR
Ms6440f	ATCACCGCGTCGTTTTTGTC	Reverse transcription -PCR
Ms6440r	ATCTTCTTGCCGGCGTAGTC	Reverse transcription -PCR
Ms6441f	CGGCCGACGTTCTACTTCTA	Reverse transcription -PCR
Ms6441r	CGTTGTCGAAGCCCGAGT	Reverse transcription -PCR
Ms2196f	TGGCCGAATCGATCAACCTC	Reverse transcription -PCR
Ms2196r	CGTTGATCCGCAAGACGATG	Reverse transcription -PCR

Notes: Restriction enzyme sites are underlined.
